# Detection and Cellular Tropism of Porcine Astrovirus Type 3 on Breeding Farms

**DOI:** 10.3390/v11111051

**Published:** 2019-11-12

**Authors:** Gaurav Rawal, Franco Matias Ferreyra, Nubia R. Macedo, Laura K. Bradner, Karen M. Harmon, Adam Mueller, Grant Allison, Daniel C.L. Linhares, Bailey L. Arruda

**Affiliations:** 1 Veterinary Diagnostic and Production Animal Medicine, College of Veterinary Medicine, Iowa State University, Ames, IA 50011-1250, USA; grawal@iastate.edu (G.R.); francomf@iastate.edu (F.M.F.); nubia@iastate.edu (N.R.M.); lbradner@iastate.edu (L.K.B.); kharmon@iastate.edu (K.M.H.); linhares@iastate.edu (D.C.L.L.); 2 Swine Services Unlimited, Inc., Rice, MN 56367, USA; dradam@swineservices.org; 3 Walcott Veterinary Clinic, Durant St. Walcott, IA 52773, USA; swinedoc2@gmail.com

**Keywords:** Porcine astrovirus type 3, astrovirus, detection, swine

## Abstract

Astroviruses cause disease in a variety of species. Yet, little is known about the epidemiology of a majority of astroviruses including porcine astrovirus type 3 (PoAstV3), which is a putative cause of polioencephalomyelitis in swine. Accordingly, a cross-sectional study was conducted on sow farms with or without reported PoAstV3-associated neurologic disease in growing pigs weaned from those farms. Additionally, a conveniently selected subset of piglets from one farm was selected for gross and histologic evaluation. The distribution of PoAstV3 in the enteric system was evaluated through in situ hybridization. PoAstV3, as detected by RT-qPCR on fecal samples, was frequently detected across sows and piglets (66–90%) on all farms (65–85%). PoAstV3 was detected subsequently at a similar detection frequency (77% vs 85%) on one farm after three months. Viral shedding, as determined by the cycle quantification value, suggests that piglets shed higher quantities of virus than adult swine. No link between gastrointestinal disease and PoAstV3 was found. However, PoAstV3 was detected by in situ in myenteric plexus neurons of piglets elucidating a possible route of spread of the virus from the gastrointestinal tract to the central nervous system. These data suggest PoAstV3 has endemic potential, is shed in the feces at greater quantities by suckling piglets when compared to sows, and infection is widespread on farms in which it is detected.

## 1. Introduction

Astroviruses are emerging viruses in the family *Astroviridae*, which is divided into two genera: *Avastrovirus* and *Mamastrovirus* [1]. Astroviruses are non-enveloped, 28–30 nm positive-sense, single stranded RNA viruses [2] that can cause disease and be detected in the feces from a wide variety of mammals and birds [3,4,5,6,7,8,9].

Porcine astroviruses (PoAstVs) are genetically diverse and are distributed worldwide [10] with five genetic lineages (PoAstV1-5), reflecting different origins, interspecies transmission and recombination events [9,11,12]. Globally, several studies have reported the molecular detection and genetic characterization of PoAstVs in various pig production systems in different countries including Canada [11,13], China [14,15,16], Croatia [17], Czech Republic [18], East Africa [19], Germany [20,21,22], Hungary [23], Italy [24], Japan [25], Slovakia [26], Sweden [27,28], South Korea [29], Thailand [30], and the United States [31,32,33,34,35,36,37]. However, the pathogenicity of a majority of PoAstVs is not well characterized with the exception of PoAstV3, which has been associated with polioencephalomyelitis in swine in both Europe [23] and the United States [31,32,33,34,35,36].

Despite the pathologic significance of PoAstV3, there is limited knowledge concerning the epidemiology and ecology of PoAstV3. Accordingly, the objective of this study was to investigate the frequency of detection, endemic potential, association of gastrointestinal disease, and tissue distribution of PoAstV3 using cross-sectional studies, pathology, and in situ hybridization on sow farms with and without reported PoAstV3-associated neurologic disease in pigs after weaning.

## 2. Materials and Methods

### 2.1. Study Design

**Figure 1:**
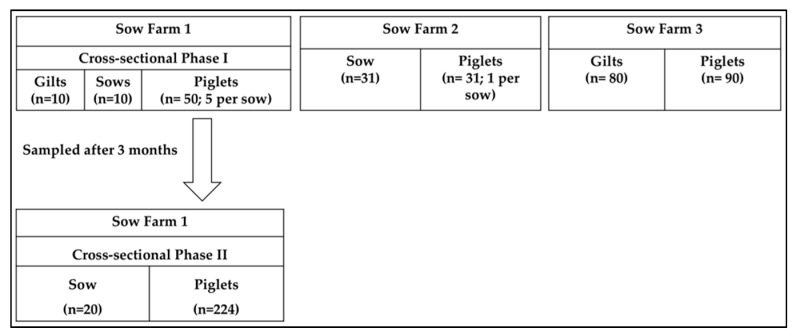
Cross-sectional study designs by farm with the number of fecal samples by pig category.

The study designs are summarized in Figure 1. Three cross-sectional studies were carried out on sow farms located in the United States without (*n* = 2; Sow Farm 1 and 2) and with (*n* = 1; Sow Farm 3) PoAstV3-associated neurologic disease in the downstream nursery. On Sow Farm 1, fecal samples were collected from ten families (sow and 5 piglets per sow) spatially distributed. In addition, ten gilts were also sampled spatially distributed in the gilt development unit (Cross-sectional Phase-I). Three months later, fecal samples were collected from twenty different families (sow and all the piglets) and spatially distributed (cross-sectional Phase-II). Parity of sows was recorded. Additionally, 13 pigs (4 controls and 9 with enteric signs) from 11 to 19 days of age were selected for gross and histologic examination from Sow Farm 1. On Sow Farm 2, fecal samples were collected from thirty-one families (sow and 1 piglet per sow) spatially distributed. On Sow Farm 3, fecal samples from all incoming gilts (*n* = 80) and conveniently selected piglets (*n* = 90) were collected.

### 2.2. Farm Selection

Sow Farm 1 reported failure-to-thrive in the nursery with detection of PoAstV3 in the feces of nursery pigs by next-generation sequencing. Sow Farm 1 is a 2200 sow farrow-to-wean farm located in Minnesota that was reported to be free of porcine reproductive and respiratory syndrome virus, *Mycoplasma hyopneumoniae*, and *Actinobacillus pleuropneumoniae*. Sow Farm 1 weaned once a week and produced roughly 25 pigs per sow per year. Sow Farm 2 was diagnosed with neurologic signs due to *Streptococcus suis* with detection of PoAstV3 RNA by RT-qPCR. Sow Farm 2 is located in Iowa. Additional information concerning Sow Farm 2 was not available at the time of this report. Sow Farm 3 had a history of PoAstV3-associated neurologic disease in nursery pigs with histologic lesions of viral polioencephalomyelitis consistently present over multiple diagnostic submissions spanning months and concurrent detection of PoAstV3 by RT-qPCR in the CNS in the absence of Teschovirus A and Sapelovirus A. Sow Farm 3 is a 3000 sow breed-to-wean farm located in southeast Iowa. The farm weans twice a week and produces roughly 26.9 pigs per sow per year. There was no reported swine movement among these three breeding farms.

### 2.3. Sample Collection

Fecal samples were collected using sterile polyester tipped swabs (Puritan^®^, Catalog no. 10805-165, Puritan Medical Products Co., ME, USA) placed in a 5-mL polystyrene round bottom tube (FALCON, Catalog no. 352054, Corning Incorporated Life Sciences, MA, USA). Each tube contained 1 mL of phosphate buffered saline (Gibco^TM^, PBS pH 7.4 (1×), Catalog no. 10010023, Grand Island, NY, USA) and samples were stored at −80 °C until testing.

### 2.4. PoAstV3 RT-qPCR

All samples were tested for the presence of PoAstV3 RNA at the Iowa State University Veterinary Diagnostic Laboratory (ISU-VDL) using standard protocols. Extraction of RNA was performed using the MagMAX™ Pathogen RNA/DNA Kit isolation kit (Life Technologies, Carlsbad, CA, USA) and a Kingfisher 96 magnetic particle processor (Thermo-Fisher Scientific, Waltham, MA, USA) using a high-volume procedure. This particular assay targets a portion of the polymerase gene specific to PoAstV3. An xeno internal positive control (XIPC) RNA template^38^ was added to the lysis solution at 50,000 copies/extraction. At least one negative extraction control (NEC) and one negative amplification control (NAC) was included in each PCR assay. NEC consisted of a well included on the extraction plate with sterile, 1X PBS substituted for sample and NAC consisted of a nuclease-free water substituted for template for one PCR reaction on the PCR plate.

Primer sequences were as follows: forward primer sequence ATGACYCTCTATGGGAAACTCCTT, reverse primer sequence GTGCCTRGCAACAACCTCCAA, and a minor groove binding probe sequence FAM-TTGGCCAYAACCTCCCTGA-MGB. The eluted RNA and the PoAstV3 and XIPC specific primers and probe were mixed with commercial reagents TaqMan^®^ Fast Virus 1-Step Master Mix (Life Technologies, Catalog no. 4444436, Foster City, CA, USA). The final concentration of the PoAstV3 primers and probe were 400nM and 200nM, respectively. The RT-qPCR reaction was conducted on the ABI 7500 Fast instrument (Life Technologies, Catalog no. 4406985, Waltham, MA, USA) in fast mode as follows: 1 cycle at 50 °C for 5 min, 1 cycle at 95 °C for 20 s, 40 cycles at 95 °C for 3 s, and 60 °C for 30 s. The results were analyzed using an automatic baseline setting with a threshold at 0.1. Cq values of less than 40 were considered positive. The equation for PoAstV3 genomic copy number per ml of sample was 10^((Cq−45.82)/−3.287)^. The standard curve is available in Supplementary Figure S1.

### 2.5. Pathology and In Situ Hybridization

Thirteen pigs (9 pigs with diarrhea and 4 pigs without diarrhea) from 11 to 19 days of age were selected for necropsy from Sow Farm 1. Gross and histologic evaluation of all piglets was performed by a veterinary diagnostic pathologist blinded to clinical presentation and all test results. Fundus of the stomach, duodenum at the level of the pancreas, two additional sections of duodenum, four to five sections of jejunum, four to five sections of ileum, cecum, two cross-sections of the spiral colon and mesenteric lymph node were fixed in 10% neutral buffered formalin, processed by standard technique and stained with hemotoxylin and eosin. Findings were recorded in the case record. Sections from these paraffin blocks were also used for RNAscope^®^ in situ hybridization (ISH).

Blocks that contained stomach, duodenum, jejunum, ileum, cecum, spiral colon, and mesenteric lymph node from four piglets were selected based on a combination of a low PoAstV3 Cq value and adequate tissue architecture for RNAscope ISH. Blocks of intestinal tissue from a pig that was known to be negative for PoAstV3 by RT-qPCR on feces were also selected for RNAscope ISH. RNAscope^®^ ISH was performed with RNAScope^®^ 2.5 HD Reagent Kit-Red (Catalog no. 322350, Advanced Cell Diagnostics, Newark, CA, USA) according to the manufacturer’s instructions as described by Matias et al., 2019. Paraffin blocks stored at room temperature (RT) were retrieved and 5 µm sections were trimmed and mounted on Superfrost^®^ Plus slides (Catalog no. 4951PLUS4, Thermo Fisher Scientific, Waltham, MA, USA). Slides were then heated for 1 h at 60 °C followed by a de-paraffinization treatment consisting of two consecutive washes of 100% xylene and two washes of 100% ethanol. Slides were treated with Pretreat 1 solution (hydrogen peroxide) at RT for 10 min and rinsed with distilled water. Slides were then immersed in target retrieval solution for 30 min at 100 °C, with subsequent protease treatment for 30 min at 40 °C. RNAScope^®^ probes targeting PoAstV3 capsid RNA (Catalog no. 51623, Advanced Cell Diagnostics, Newark, CA, USA), RNAScope^®^ positive control probe Sc-PPIB targeting swine PPIB gene (Catalog no. 428591, Advanced Cell Diagnostics, Newark, CA, USA), and RNAScope^®^ negative control probe DapB (Catalog no. 310043, Advanced Cell Diagnostics, Newark, CA, USA) were designed and synthesized by Advanced Cell Diagnostics. Probes were then hybridized for 2 h at 40 °C. Six rounds of amplification were performed (solution kit Amp1-6) followed by the incubation with red chromogenic detection solution for 10 min at RT. Slides were counterstained with 50% hematoxylin solution for 2 min, rinsed in a 0.02% ammonia solution for 10 s and then washed 5 times in distilled water. Slides were then dried at 60 °C for 15 min and cooled down at RT for 5 min. Finally, slides were submerged into a 100% xylene solution and immediately cover slipped with EcoMount solution (Catalog no. EM897L, Biocare Medical, Pacheco, CA, USA) and tissue sections examined using an Olympus BX43 bright-field microscope (Olympus Corporation, Tokyo, Japan).

### 2.6. Statistical Analysis

Statistical analysis was conducted with SAS 9.4 software (SAS Institute Inc., Cary, NC, USA) with a level of significance of 0.05. MS-Excel was used to manage RT-qPCR data for information regarding detection frequencies, mean and range of Cq values. Analysis of Variance (ANOVA) along with multiple comparison Tukey test was used to compare the Cq means in Sow Farm 1 (Phase-I) and was used to compare frequency of detection across Sow Farms 1, 2 and 3 and between cross-sectional Phase-I and Phase-II on Sow Farm 1. Binomial regression analysis was used to measure the association between litter status, i.e., sow status and their corresponding piglets in Sow Farm 1 (Phase-I and Phase-II) and Sow Farm 2. A litter was considered positive when PoAstV3 was detected by RT-qPCR in ≥50% of piglets tested. The McNemar’s test was used to assess a link between detection of PoAstV3 by RT-qPCR in feces and histologic lesions. As the equal variance assumption was rejected using test for equality of variance, a Welch–Satterthwaite *t*-test was used to determine whether there is a significant difference in the Cq means between sows and piglets in Sow Farm 1 (Phase-II), Sow Farm 2 and between gilts and piglets in Sow Farm 3 and the Analysis of Variance (ANOVA) was used to compare the Cq means in Sow Farm 1 (Phase-I).

## 3. Results

### 3.1. Sow Farm 1(Cross-Sectional Phase-I)

The frequency of detection of PoAstV3 was 77% (54 of 70) by RT-qPCR with varying levels of detection across different age groups (Table 1). The distribution of the number of samples in a given Cq range as well as the corresponding gc/mL of sample range is provided in Supplemental Table S1. The frequency of PoAstV3 in sows or piglets was significantly greater than in gilts (*p* < 0.0001), with no significant difference in detection between sows and piglets (*p* = 0.7471). The mean Cq values of gilts and sows compared to piglets were significantly higher (*p* < 0.0001 and *p* = 0.0241, respectively). The association between sow status and piglet status by RT-qPCR was not statistically significant (*p* = 0.4766).

### 3.2. Sow Farm 1(Cross-Sectional Phase-II)

After three months, the overall detection of PoAstV3 was found to be 85% (207 of 244) by RT-qPCR with varying levels of detection across different age groups (Table 1). There was no statistical difference found in the overall detection of PoAstV3 on Sow Farm 1 between Phase-I and Phase-II (*p* = 0.1479). The mean PoAstV3 RT-qPCR Cq value of piglets was significantly lower than the mean Cq value of sows (*p* < 0.0001). Six piglet fecal samples (3%) contained 9.66–1.18 × 10^9^ gc/mL of the sample (Supplementary Table S1). The association between sow status and piglet status by RT-qPCR was not statistically significant at *p* = 0.2293. PoAstV3 was detected in sows across sampled parities with the exception of parity 10 (Table 2).

#### Pathology and ISH

Pathology and ISH results are summarized in Table 3. PoAstV3 was detected by RT-qPCR in fecal samples from 9 of 13 piglets (69%; mean Cq = 26.02; Cq range = 17.96–35.71). PoAstV3 was detected in all controls (*n* = 4; mean Cq=21.33) and five of the piglets with clinical enteric disease (56%; mean Cq = 29.78). Fluid filled small and large intestine was noted in 25% and 67% of piglets in the control and enteric disease group, respectively. Gastritis characterized by three or more of the following: gastric pit elongation; loss of parietal cells, mucus neck cells and surface mucous cells; aggregates of inflammatory infiltrates in the lamina propria; and/or erosion and ulceration was observed in 25% (1 of 4) and 56% (5 of 9) of piglets in the control and enteric disease group, respectively. Atrophic enteritis characterized by segmental blunting and fusion of villi and colitis characterized by two or more of the following: crypt elongation, inflammatory infiltrates in the lamina propria and/or submucosa, and/or erosion were observed in 100% and 50% of piglets in the control group, respectively. Atrophic enteritis and colitis were both observed in 33% of piglets in the enteric disease group. There was no statistical difference found between detection of PoAstV3 by RT- qPCR in feces with histologic evidence of gastritis (*p* = 0.2568), atrophic enteritis (*p* = 0.4142) or colitis (*p* = 0.1025).

Bacterial culture of enteric tissue from two piglets with neutrophils in the lamina propria resulted in a heavy growth of *Clostridium perfringens* from one pig and high growth of smooth/mucoid *Escherichia coli* from the other. Coccidia was observed in a single pig from the enteric disease group. Rotavirus group A, rotavirus group B, rotavirus group C, delta coronavirus, transmissible gastroenteritis virus, and porcine epidemic diarrhea virus were not detected by RT-qPCR on fecal samples.

**Figure 2:**
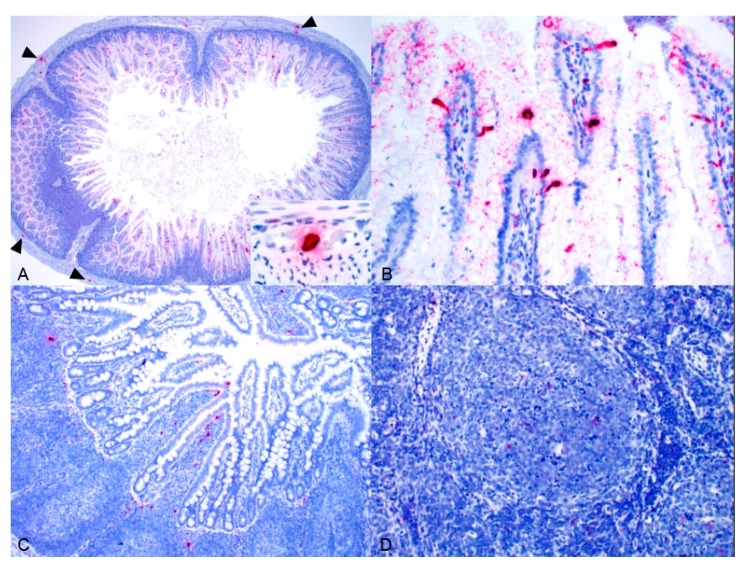
PoAstV3 detected by ISH (red labeling) in the enterocytes and myenteric plexus neurons (arrowhead and inset) of the jejunum of Pig 4 (**A**), enterocytes and lamina propria of the jejunum of Pig 13 (**B**), enterocytes, lamina propria and Peyer’s patches of the ileum of Pig 10 (**C**), and germinal center and mantel zone of the mesenteric lymph node of Pig 4 (**D**).

PoAstV3 was detected via ISH in all PoAstV3-positive animals assayed (*n* = 4) with variable levels of labeling at both the pig and tissue level as shown in Figure 2. PoAstV3 was detected in the myenteric plexus neurons of the ileum and less commonly the jejunum in three of the four pigs assayed (Figure 2A). Labeling was present in rare to occasional gastric pit cells in three of the four pigs assayed, rare to multiple enterocytes of the jejunum in three of four pigs and the lamina propria of the jejunum in a single pig (Figure 2A,B), and rare to occasional enterocytes of the ileum in three of the four pigs as well as the lamina propria (*n* = 1) and Peyer’s patches (*n* = 2; Figure 2C). PoAstV3 was detected in the mesenteric lymph node of all pigs with labeling present in the germinal center, periarticular lymphoid sheets and mantel zone (Figure 2D). PoAstV3 was not detected in the duodenum or cecum of any pig assayed. PoAstV3 was not detected via ISH in the intestinal in the known PoAstV3-negative pig (Supplemental Figure S2).

### 3.3. Sow Farm 2

PoAstV3 was detected in 74% (46 of 62; mean Cq = 29.76; Cq range= 14.69–38.66) of fecal samples by RT-qPCR (Table 1). The distribution of the number of samples in a given Cq range is provided in Supplemental Table S1. The frequency of detection was 97% (30 of 31; mean Cq = 26.50; Cq range = 14.69–38.66) in piglets and 52% (16 of 31; mean Cq = 35.89; Cq range= 30.99–38.02) in sows. PoAstV3 detection between sows and piglets and the Cq values between sows and piglets were significantly different (*p* < 0.0001). Two piglet fecal samples (3%) contained 9.66–1.18 × 10^9^ gc/mL of sample. The association between sow status and piglet status by RT-qPCR was not statistically significant (*p* = 0.99).

### 3.4. Sow Farm 3

PoAstV3 was detected in 100% (90 of 90; mean Cq = 31.70; Cq range = 30.74 to 32.68) of fecal samples from piglets by RT-qPCR (Table 1). PoAstV3 was detected in 28.75% (23 of 80; mean Cq = 32.00; Cq range = 28.16 to 39.09) of fecal samples from gilts. Statistical analysis showed a significant difference in PoAstV3 detection between piglets and gilts (*p* < 0.0001). The mean Cq value between gilts and piglets was not statistically significant (*p* = 0.6187).

## 4. Discussion

Astroviruses have been detected in the feces of a wide variety of mammals and birds [3,4,5,6,7,8,9] and have been associated with neurologic disease in multiple species including swine over the last several years [23,27,31,32,34,35,36,38,39,40,41,42]. Despite this pathologic potential, there is much to learn concerning the epidemiology, ecology and pathophysiology of astroviruses including PoAstV3. The objectives of this study were to investigate the frequency of detection, endemic potential, association of gastrointestinal disease, and cellular tropisms of PoAstV3 using cross-sectional studies, pathology, and in situ hybridization on sow farms with and without reported PoAstV3-associated neurologic disease in pigs after weaning.

PoAstV3 was commonly detected in fecal samples on all three farms sampled regardless of reported PoAstV3-associated neurologic disease status in growing pigs originating and weaned from those farms. This data suggests that when detected on a farm, PoAstV3 is likely being shed in the feces of numerous sows and piglets; however, only a fraction of those infected develop clinical disease. PoAstV3 was detected in the billions of gc/mL of sample in the feces of a small subset of piglets from both Sow Farm 1 and Sow Farm 2. Based on the RT-qPCR results of this study, both sows and piglets commonly shed PoAstV3, but piglets on average shed significantly more virus in their feces than sows.

The statistical analysis showed no association in litter status of Sow Farm 1 (Phase-I and Phase-II) and Sow Farm 2 indicating that piglet status cannot be predicted by their sow status using RT-qPCR. It is likely other factors including sow immunity may explain this discordant finding. PoAstV3 was shed by all parities of sows with the exception of the only two parity 10 sows sampled, which were negative. Although immunity may develop, sample size limits any conclusions.

PoAstV3 was also found at a similar detection (77% compared to 85%) after three months on one farm suggesting PoAstV3 has endemic potential. This is not surprising as AstVs are non-enveloped and hardy in the environment [43]. AstVs are resistant to inactivation by acidic pH, heat, many detergents, and lipid solvents [44]. Free chlorine disinfection was much less efficient at inactivating astrovirus than poliovirus [45]. Viral infectivity of AstVs is decreased two-fold when exposed to pH 3.0 for 3 h compared to pH 4.0 for 3 h [45].

In the subset of piglets from Sow Farm 1 Phase-II that underwent gross and histologic evaluation, PoAstV3 was detected by RT-qPCR in a majority of piglets but at a lower mean Cq value in control pigs. There was no statistically significant association between PoAstV3 detection by RT-qPCR and gastritis, atrophic enteritis, or colitis. PoAstV3 was not detected in attenuated enterocytes in pigs with atrophic enteritis by ISH but was commonly detected in enterocytes that were histologically normal. The absence of histologic changes of infected cells may be a result of the mechanism(s) of virus release as it is thought to occur in the absence of cell death [46] through a non-lytic mechanism [47]. PoAstV3 was detected by ISH in the myenteric plexus neurons of three out of the four pigs assayed elucidating a possible mode of transport of PoAstV3 from the enteric system to the central nervous system. Much like poliomyelitis due to poliovirus in humans [48,49,50], it appears based on the data presented in this report that a small subset of infected pigs developed polioencephalomyelitis following PoAstV3 infection. This may be a result of the peripheral nervous system barriers that include limited replication of the virus in peripheral neurons, inefficient retrograde axonal transport and type I interferon response [50].

Identification of risk factors associated with PoAstV3-associated neurologic disease requires additional investigation but could include exposure dose, genetic determinants, intestinal microbiota and/or immune suppression as a result of co-infections, co-morbidities, and stress. Further studies are also needed to understand the infection dynamics and antibody response over time, comparative sensitivity of sample types, and efficacy of commercial disinfectants. This information would provide foundational knowledge required to prevent and control disease.

## Figures and Tables

**Table 1:** Distribution of detection of porcine astrovirus type 3 (PoAstV3) in fecal samples across various age groups across farms by RT-qPCR.

Age Group	Sow Farm 1 (Phase-I)		Sow Farm 1 (Phase-II)		Sow Farm 2		Sow Farm 3	
	% Positive (Sample *n*)	Mean (Cq Range)	% Positive (Sample *n*)	Mean (Cq Range)	% Positive (Sample *n*)	Mean (Cq Range)	% Positive (Sample *n*)	Mean (Cq Range)
Sows	90% ^a^ (9 of 10)	35.17 ^a^ (32.47– 39.43)	75% ^a^ (15 of 20)	34.23 ^a^ (31.05–38.05)	52% ^a^ (16 of 31)	35.89 ^a^ (30.99–38.02)		
Gilts	20% ^b^ (2 of 10)	37.01 ^a^ (35.71– 38.32)					29% ^a^ (23 of 80)	32.00 ^a^ (28.16– 39.09)
Piglets	86% ^a^ (43 of 50)	31.82 ^b^ (22.19–38.96)	86% ^a^ (192 of 224)	26.86 ^b^ (14.60–39.15)	97% ^b^ (30 of 31)	26.50 ^b^ (14.69–38.66)	100% ^b^ (90 of 90)	31.70 ^a^ (30.74–32.68)

^a,b^ Different letters indicate significant difference between the age groups by individual farm. Cq = cycle quantification value.

**Table 2:** Distribution of detection of PoAstV3 in fecal samples of sows across different parities by RT-qPCR.

Parity	% Positive (Sampling *n*)	Mean (Cq Range)
1	88% (7 of 8)	33.72 (31.05–36.93)
4	67% (4 of 6)	34.17 (32.68–37.54)
5	100% (2 of 2)	36.58 (35.11–38.04)
7	100% (2 of 2)	33.75 (33.05–34.45)
10	0% (0 of 2)	-

Cq = cycle quantification value.

**Table 3:** Summary of the pathology and RNAscope in situ hybridization of tissues collected from subset of piglets from Sow Farm 1 Phase-II.

Pig ID	Group	Age (days)	Litter ID	GL	PoAstV3 Cq	GS	AE	CL	In Situ Hybridization							
									GM	Duo	Jej	Ile	CM	SC	MSLN	MP
1	Control	11	A	DU	17.96	0	1	0	NA	NA	NA	NA	NA	NA	NA	NA
2	Control	11	A	FF	25.42	0	1	0	ND	ND	ND	OE	ND	OC	GC;PLS;M	ND
3	Control	11	B	DU	21.10	1	1	1	NA	NA	NA	NA	NA	NA	NA	NA
4	Control	11	C	DU	20.83	0	1	1	OGP	ND	RE-NE	ND	ND	RC;L;CL	GC;PLS;M	I;J
5	Enteric	11	A	DU	UD	1	0	0	NA	NA	NA	NA	NA	NA	NA	NA
6	Enteric	15	A	FF	32.25	1	0	1	NA	NA	NA	NA	NA	NA	NA	NA
7	Enteric	11	B	FF	31.66	0	0	0	NA	NA	NA	NA	NA	NA	NA	NA
8	Enteric	15	B	FF	UD	1	0	0	NA	NA	NA	NA	NA	NA	NA	NA
9	Enteric	15	C	FF	UD	0	1	1	NA	NA	NA	NA	NA	NA	NA	NA
10	Enteric	11	C	DU	23.50	1	0	0	RGP	ND	RE	OE;LP;PP	ND	ND	GC;PLS;M	I
11	Enteric	11	D	FF	UD	0	1	0	NA	NA	NA	NA	NA	NA	NA	NA
12	Enteric	19	D	DU	35.71	0	1	1	NA	NA	NA	NA	NA	NA	NA	NA
13	Enteric	15	E	FF	25.80	1	0	0	RGP	ND	ME;LP	RE;PP	ND	ND	PLS	I

GL = gross lesions; Cq = PoAstV3 cycle quantification value; GS = gastritis; AE = atrophic enteritis; CL = colitis; 0 = absent; 1 = present; GM = gastric mucosa; Duo = duodenum; Jej = jejunum; Ile = ileum; CM = caecum; SC = spiral colon; MSLN = mesenteric lymph node; MP = myenteric plexus; DU = diagnostically unremarkable; UD = undetected after 40 cycles; FF = fluid filled small and large intestines; NA = not available; ND = not detected; OGP = occasional gastric pit cells; RGP = rare gastric pit cells; RE = rare enterocytes; NE = numerous enterocytes; ME = multiple enterocytes; LP = lamina propria; OE = occasional enterocytes; PP = Peyer’s patches; OC = occasional colonocytes; RC = rare colonocytes; L = lumen; CL = crypt lumen; GC = germinal centers; PLS = periarteriole lymphoid sheets; M = mantel zone; I = myenteric plexus neurons of the ileum; J = myenteric plexus neurons of the jejunum.

## Supplementary Materials

The following are available online at https://www.mdpi.com/1999-4915/11/11/1051/s1, Figure S1: PoAstV3 RT-qPCR standard curve; Figure S2: PoAstV3 ISH in the jejunum of a pig that was known to be negative for PoAstV3 by RT-qPCR. The absence of red labeling illustrates the specificity of the PoAstV3 ISH; Table S1: Quantification of PoAstV3 in fecal samples using RT-qPCR.
