# Supplementary material for: Detection and Cellular Tropism of Porcine Astrovirus Type 3 on Breeding Farms
Source: Viruses. 2019 Nov 12;11(11):1051. doi: 10.3390/v11111051 (PMC6893673; doi:10.3390/v11111051)
Supplement: Supplementary file 1 [file viruses-11-01051-s001.zip › Supplemetary Table and Figures/Supplementary Figure 2.pdf]

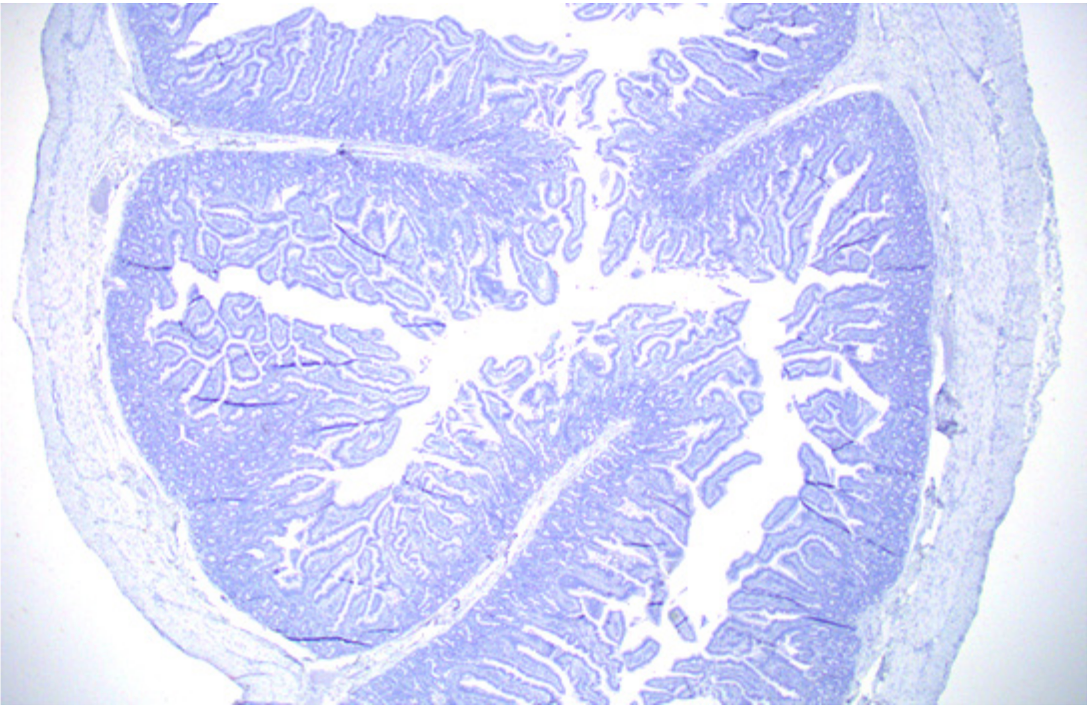

Supplementary Figure 2. PoAstV3 ISH in the jejunum of a pig that was known to be negative for PoAstV3 by RT-qPCR. The absence of red labeling illustrates the specificity of the PoAstV3 ISH.
