# Supplementary material for: Detection and Cellular Tropism of Porcine Astrovirus Type 3 on Breeding Farms
Source: Viruses. 2019 Nov 12;11(11):1051. doi: 10.3390/v11111051 (PMC6893673; doi:10.3390/v11111051)
Supplement: Supplementary file 1 [file viruses-11-01051-s001.zip › Supplemetary Table and Figures/Supplementary Table 1..docx]

**Supplementary Table 1.** Quantification of PoAstV3 in fecal samples using RT-qPCR.

| Cq range | GC/ml of sample* | Sow Farm 1 (Phase-I) | Sow Farm 1 (Phase-II) | Sow Farm 2 | Sow Farm 3 |
| --- | --- | --- | --- | --- | --- |
| 10-12 | 7.90-1.95 X 10^10^ | 0% (0/70) | 0% (0/244) | 0% (0/62) | 0% (0/170) |
| 13-16 | 9.66-1.18 X 10^9^ | 0% (0/70) | 3% (6/244) | 3% (2/62) | 0% (0/170) |
| 17-19 | 5.86-1.44 X 10^8^ | 0% (0/70) | 7% (18/244) | 7% (4/62) | 0% (0/170) |
| 20-22 | 7.17-1.77 X 10^7^ | 1% (1/70) | 16% (39/244) | 11% (7/62) | 0% (0/170) |
| 23-26 | 8.76-1.07 X 10^6^ | 12% (8/70) | 18% (45/244) | 8% (5/62) | 0% (0/170) |
| 27-29 | 5.32-1.31 X 10^5^ | 13% (9/70) | 10% (25/244) | 2% (1/62) | 2% (4/170) |
| 30-32 | 6.51-1.60 X 10^4^ | 10% (7/70) | 9% (22/244) | 11% (7/62) | 61% (103/170) |
| 33-36 | 7.95-0.97 X 10^3^ | 21% (15/70) | 14% (34/244) | 19% (12/62) | 2% (3/170) |
| 37-39 | 4.82-1.19 X 10^2^ | 20% (14/70) | 8% (18/244) | 13% (8/62) | 2% (3/170) |
| 40 | 5.90 X 10^1^ | 23% (16/70) | 15% (37/244) | 26% (16/62) | 33% (57/170) |

*10^((Cq−45.82)/−3.287)^. GC= genomic copies.
