# Supplementary figures and images for: Detection and Cellular Tropism of Porcine Astrovirus Type 3 on Breeding Farms
Source: Viruses. 2019 Nov 12;11(11):1051. doi: 10.3390/v11111051 (PMC6893673; doi:10.3390/v11111051)

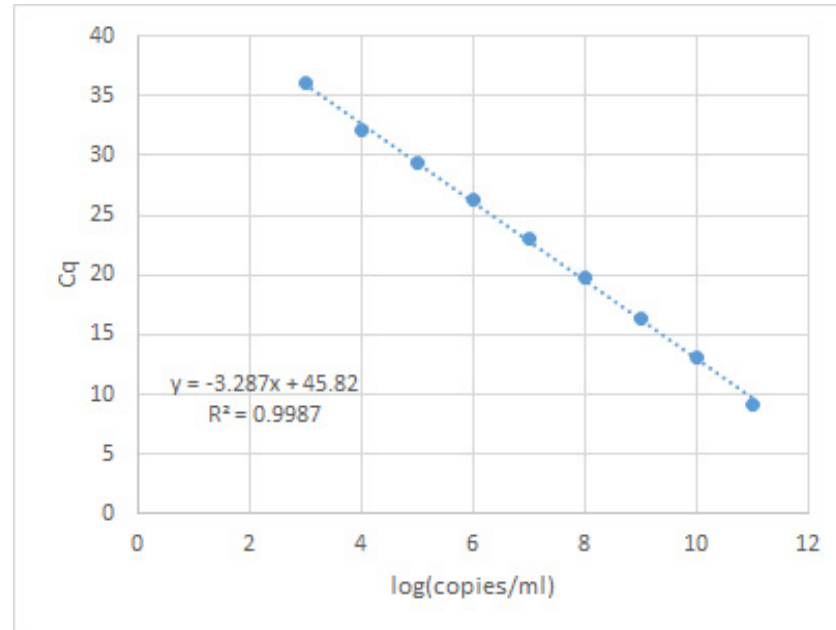

Supplementary Figure 1. PoAstV3 RT-qPCR standard curve.

Supplement: Supplementary file 1 [file viruses-11-01051-s001.zip › Supplemetary Table and Figures/Supplementary Figure 1.pdf]
